# The Coronary Sinus Reducer for refractory angina

**DOI:** 10.1007/s12471-021-01557-8

**Published:** 2021-03-06

**Authors:** P. Damman, J. J. Piek

**Affiliations:** 1 Department of Cardiology, Radboud University Medical Center, Nijmegen, The Netherlands; 2 Department of Cardiology, Heart Center, Amsterdam Cardiovascular Sciences, University of Amsterdam, Amsterdam UMC, Amsterdam, The Netherlands

Despite progress in revascularisation strategies and techniques for coronary artery disease, up to 10% of patients have refractory angina [1]. Refractory angina is defined as long-lasting symptoms (3 months or longer) due to established reversible ischaemia in the presence of obstructive coronary artery disease (CAD), which cannot be controlled by escalating medical therapy or revascularisation [2]. Refractory angina is associated with a poor quality of life, frequent hospitalisations and a high level of resource utilisation [2]. The incidence is expected to rise due to more advanced CAD, multiple comorbidities and ageing of the population.

According to the recent European Society of Cardiology (ESC) guidelines on chronic coronary syndrome, there are multiple potential treatment options, including external counter pulsation, extracorporeal shockwave therapy, neuromodulation, gene therapy, autologous cell therapy and coronary sinus constriction.

With regards to coronary sinus constriction, the exact physiological mechanism is unclear. The rationale of coronary sinus constriction is based on the development of an upstream pressure gradient that results in the redistribution of blood flow from the less ischaemic epicardium towards the ischaemic endocardium [3]. This can be achieved by using the Coronary Sinus Reducer System (CSRS), a metal mesh device that is premounted on a balloon catheter and has an hourglass shape after balloon dilatation. After placement of the device in the coronary sinus, local flow disruption and vascular reaction lead to a hyperplastic response in the vessel wall, with occlusion of the fenestrations in the metal mesh. The central orifice of the device remains patent and becomes the sole path for blood flow through the coronary sinus, ultimately creating a pressure gradient.

The CSRS has been shown to improve symptoms and quality of life in patients with refractory angina in the small CORSIRA trial [4]. In CORSIRA, 104 patients with Canadian Cardiovascular Society (CCS) class III or IV angina and myocardial ischaemia, who were not candidates for revascularisation, were randomised to the CSRS or a sham procedure. At 6‑month follow-up, 35% of the CSRS patients as compared with 15% in the control group showed a significant improvement of more than two CCS angina classes. Based on the CORSIRA trial, the ESC guideline recommends that CSRS “may be considered” for refractory angina [2].

In addition to the randomised CORSIRA trial, the current issue includes important data from a real-world population of patients undergoing CSRS implantation at the University Medical Centre Utrecht and the St. Antonius Hospital in Nieuwegein [5]. Registry data provide valuable and complementary evidence to randomised clinical trials, that typically includes a selection of low-risk patients. For example, patients with a left ventricular ejection fraction of less then 30% were not included in the CORSIRA trial.

In the present multicentre registry, a total of 132 patients were included who were treated between 2014 and 2020. The registry concerns a patient population with extensive coronary artery disease not suitable for revascularisation, who were treated previously with PCI (83%) or CABG (76%). More than 40% of patients were treated with > 3 antianginal agents, compared with 5% of the patients in the CORSIRA trial. Despite these differences, the results of this registry were similar to the CORSIRA trial, 67% of the patients showed an improvement of at least 1 CCS class and 34% of at least 2 CCS classes. More importantly, no safety concerns evolved. The CSRS was successfully implanted in 99% of the patients and only minor complications occurred. Furthermore, no ischaemic events occurred at long-term follow-up after the recommended 3‑month treatment with clopidogrel. This is an important finding, considering the potential prothrombotic environment of implanting a large device in a low-flow vasculature of the coronary sinus.

The authors are to be congratulated with this report, providing additional evidence on a treatment option in patients with refractory angina, an area in which larger and large randomised clinical trials and registries are required to define the role of this novel device. Despite the promising results of this registry, some aspects require additional comments.

A large proportion of non-responders remain. Potential explanations for this non-responsiveness include incomplete endothelialisation of the CSRS resulting in inadequate pressure gradient induction across the device. There are currently no periprocedural and procedural measurements available to better predict device success and responders or non-responders. Another explanation might be an epicardial cause for the anginal complaints, either obstructive or non-obstructive. Coronary vasomotion disorders of epicardial arteries and microcirculatory dysfunction have been estimated to be present in 50% of patients with refractory angina after PCI [6]. The authors state that in some “no-option” patients, evidence of ischaemia was not present, suggesting that underlying dynamic vasomotor dysfunction may have caused symptoms. Finally, as the authors and previous groups have stated, the exact physiological mechanism for CSRS benefit remains unclear. Additional research is mandatory for better identification of responders and non-responders as well as the predictors of responsiveness and non-responsiveness. One potential predictor or outcome would be myocardial ischaemia, although there is currently no evidence of a reduction of ischaemia after CSRS. Moreover, a placebo effect cannot be ruled out to explain a part of the beneficial effect of CSRS. This is endorsed by the COSIRA trial, in which a substantial improvement in angina symptoms was observed in 42% of the sham-controlled group.

In conclusion, the CSRS is a safe and simple treatment option for refractory angina and a promising technique, but its potential is still poorly defined including a comparison with alternative treatment options for refractory angina.
